# Zika virus threshold determines transmission by European *Aedes albopictus* mosquitoes

**DOI:** 10.1080/22221751.2019.1689797

**Published:** 2019-11-18

**Authors:** Marie Vazeille, Yoann Madec, Laurence Mousson, Rachel Bellone, Hélène Barré-Cardi, Carla Alexandra Sousa, Davy Jiolle, André Yébakima, Xavier de Lamballerie, Anna-Bella Failloux

**Affiliations:** a Institut Pasteur, Department of Virology, Arboviruses and Insect Vectors, Paris, France; b Institut Pasteur, Department of Infection and Epidemiology, Emerging Diseases Epidemiology, France; c Office de l’Environnement de la Corse, Observatoire Conservatoire des Insectes de Corse, Corte, France; d Global Health and Tropical Medicine, Instituto de Higiene e Medicina Tropical, Universidade Nova de Lisboa, Lisboa, Portugal; e UMR MIVEGEC (IRD 224-CNRS 5290-UM), Maladies Infectieuses et Vecteurs: Ecologie, Génétique, Evolution et Contrôle, Institut de Recherche pour le Développement (IRD), Montpellier, France; f VECCOTRA, Rivière salée, Martinique; g Unité des Virus Emergents (UVE), Aix Marseille Université, IHU Méditerranée Infection, Marseille, France

**Keywords:** Zika, epidemic potential, arbovirus, *Aedes albopictus*, Europe

## Abstract

Since its emergence in Yap Island in 2007, Zika virus (ZIKV) has affected all continents except Europe. Despite the hundreds of cases imported to European countries from ZIKV-infested regions, no local cases have been reported in localities where the ZIKV-competent mosquito *Aedes albopictus* is well established. Here we analysed the vector competence of European *Aedes* (*aegypti* and *albopictus*) mosquitoes to different genotypes of ZIKV. We demonstrate that *Ae. albopictus* from France was less susceptible to the Asian ZIKV than to the African ZIKV. Critically we show that effective crossing of anatomical barriers (midgut and salivary glands) after an infectious blood meal depends on a viral load threshold to trigger: (i) viral dissemination from the midgut to infect mosquito internal organs and (ii) viral transmission from the saliva to infect a vertebrate host. A viral load in body ≥4800 viral copies triggered dissemination and ≥12,000 viral copies set out transmission. Only 27.3% and 18.2% of *Ae. albopictus* Montpellier mosquitoes meet respectively these two criteria. Collectively, these compelling results stress the poor ability of *Ae. albopictus* to sustain a local transmission of ZIKV in Europe and provide a promising tool to evaluate the risk of ZIKV transmission in future outbreaks.

## Introduction

Before the WHO considered it as a public health emergency of international concern in February 2016 [1], Zika virus (ZIKV, *Flavivirus*, *Flaviviridae*) was globally a neglected mosquito-borne virus. First identified in Uganda [2], ZIKV circulates primarily within a sylvatic cycle transmitted between *Aedes* mosquitoes and non-human primates [3]. Urban ZIKV outbreaks were associated with human-biting mosquitoes, mainly *Aedes aegypti* in tropical regions and presumably, *Aedes albopictus* in both tropical and temperate countries [4]. After a first epidemic in Yap Island in 2007 [5], a large ZIKV outbreak took place in French Polynesia in 2013–2014, and spread to other Pacific Islands [6]. In early 2015, ZIKV hit Brazil [7] and spread rapidly across the Americas causing hundreds of thousands of ZIKV disease cases, some associated with unusual severe clinical symptoms, microcephaly in newborns and neurological disorders [8]. Phylogenetic analysis indicated that the circulating ZIKV belonged to the Asian genotype [9,10]. On November 2016, the end of Zika alert was declared with a significant decline in ZIKV cases including congenital Zika syndrome [11]. However, the virus continues to spread in regions where competent vectors reside. Surprisingly, despite hundreds of imported cases in Europe (21 European countries, 2,133 cases [12]) and rapid expansion of the potential ZIKV vector *Ae. albopictus* [13], no locally acquired vector-borne cases were reported. Nearly 43% of imported cases were returning in regions where *Ae. albopictus* was established, most cases from the Caribbean (Guadeloupe, Martinique and the Dominican Republic; [12]). First established in Europe since 1979 [14] and more massively since 1990 [15], *Ae. albopictus* is regarded as experimentally competent to ZIKV since the virus replicates, disseminates and transmits with viruses excreted in saliva [16]. Nevertheless, European *Ae. albopictus* was less efficient to ZIKV than *Ae. aegypti* [17–20]. To date, the arguments supporting the lack of local ZIKV cases in Europe during the Zika pandemic are unconvincing. Against all expectations, in October 2019, two autochthonous cases of Zika have been reported in the south of France and to date, the viral genotype involved remains unknown (https://www.santepubliquefrance.fr/maladies-et-traumatismes/maladies-a-transmission-vectorielle/chikungunya/articles/donnees-en-france-metropolitaine/chikungunya-dengue-et-zika-donnees-de-la-surveillance-renforcee-en-france-metropolitaine-en-2019). Here, we estimate accurately the vector competence of *Ae. albopictus* for ZIKV and evaluate the threshold value of viral copies needed to trigger dissemination and transmission in the mosquito vector. We show that a viral load in body ≥4800 viral copies allows dissemination and ≥12,000 copies enable transmission. Only a limited number of *Ae. albopictus* mosquitoes meet these two criteria compared to *Ae. aegypti*.

## Materials and methods

### Ethic statements

Animals were housed in the Institut Pasteur animal facilities accredited by the French Ministry of Agriculture for performing experiments on live rodents. Work on animals was performed in compliance with French and European regulations on care and protection of laboratory animals (EC Directive 2010/63, French Law 2013–118, 6 February 2013). All experiments were approved by the Ethics Committee #89 and registered under the reference APAFIS#6573–201606l412077987 v2.

### Mosquito populations

Four populations were collected using ovitraps and eggs were shipped to the Institut Pasteur in Paris (France): 2 *Ae. aegypti* (AA; FUNCHAL (Madeira, F0, collected in 10/2017), HAITI (Port au Prince, F1, 03/2017)) and 2 *Ae. albopictus* (AL; CORSICA (Bastia, F0, 08/2017), MONTPELLIER (France, F0, 07/2018)). After immersion of eggs for 24 h in water, larvae were distributed by 200 in 1 L of dechlorinated water supplemented with 1 tablet of yeast renewed every 3 days. Pupae were daily collected and placed in a cage for adult emergence. Adults were maintained in controlled conditions (28°±1°C with a 12 h light regime, 80% relative humidity) and fed ad libitum with a 10% sucrose solution. The F0–F1 generation of mosquitoes was used for infection assays.

### ZIKV strains

Three ZIKV strains were used: two provided by EVAg (https://www.european-virus-archive.com/; ZIKV Dakar isolated from mosquitoes in 1984 (UVE/ZIKV/1984/SN/Dakar ArD 411662, African genotype, passage 4, Genbank reference: KU955592) and ZIKV Martinique isolated from a human case in 2015 (MRS_OPY_Martinique_PaRi_2015, Asian genotype, passage 3, Genbank reference: KU647676)) and ZIKV Cambodia isolated from a human case in 2010 (FSS 13025, Asian genotype, passage 3, Genbank reference: KU955593) provided by the World Reference Center for Emerging Viruses and Arboviruses (WRCEVA), University of Texas Medical Branch.

Viral stocks were prepared after 1–2 passages of isolates on Vero CCL-81 cells (ATCC®, VI, USA) maintained at 37°C. Once cytopathic effect was detected (48–72 h after infection depending on ZIKV strain), supernatants were collected and adjusted to 10% Fetal Bovine Serum (Life Technologies®, CA, USA). The virus stock was divided into 1 mL aliquots and stored at – 80 °C until use. The viral titer was estimated by serial 10-fold dilutions on Vero cells expressed in TCID_50_/mL.

### Infections of mosquitoes with ZIKV

For each combination of virus strain and mosquito population, 4–6 boxes of 60 7-day-old females were exposed to a blood meal containing 1.4 mL of washed rabbit erythrocytes and 700 μL of viral suspension supplemented with a phagostimulant (ATP) at a final concentration of 5 mM. The titer of infectious blood-meals was 10^7^ TCID_50_/mL. After 1 h, engorged females were isolated in containers and fed with 10% sucrose in an incubator maintained at 28°±1°C, a 12 h light regime and 80% humidity.

### Vector competence indices

Batches of mosquitoes were analysed at 7, 14 and 21 dpi. After cold anesthesia, individual mosquitoes were rid of their wings and legs and the proboscis was inserted into a 20 µL tip containing 5 µL of FBS. After 30 min, FBS containing saliva was collected in 45 µL of DMEM medium (Gibco, MA, USA). Abdomen and thorax, and head were separately homogenized in 300 µL of DMEM medium supplemented with 2% FBS and centrifuged at 10,000 g for 5 min. Abdomen plus thorax, head, and saliva were titrated to estimate infection, dissemination and transmission, respectively.

Infection rate (IR) is the proportion of mosquitoes with infected body (abdomen plus thorax) among examined mosquitoes; mosquitoes are able to replicate the virus in the midgut epithelial cells. Dissemination rate (DR) is the proportion of mosquitoes having virus detected in the head among mosquitoes with infected body; midgut-infected mosquitoes are able to disseminate the virus beyond the midgut and infect secondary organs/tissues through the hemolymph. Lastly, transmission rate (TR) is the proportion of mosquitoes with virus detected in saliva among mosquitoes with infected head; mosquitoes having successfully disseminated the virus are able to infect the salivary glands and to excrete virus with saliva delivered.

### Viral titration

Samples of bodies and heads were inoculated onto monolayers of Vero cells in 96-well plates and incubated for 7 days at 37°C then stained with a solution of crystal violet (0.2% in 10% formaldehyde and 20% ethanol). Presence of viral copies was asserted by observation of CPE. Saliva, heads and bodies from mosquitoes infected with ZIKV Martinique were titrated on monolayer of Vero cells in 6 well plates incubated 7 days under an agarose overlay. Sample titers were expressed as pfu (plaque-forming unit).

### Statistical analysis

Rates (infection, dissemination and transmission) were described using median and inter-quartile range (IQR). The effect of population, ZIKV strain, dpi and virus on rates was investigated using logistic regression models. Two-by-two interaction between population, ZIKV strain and dpi was systematically investigated. Viral load level was compared between groups using a student *t*-test. ROC (Receiver Operating Characteristic) curves were used to evaluate the ability of viral load level to discriminate between mosquitoes with and without dissemination and transmission. ROC curves are graphical plots that represent the ability of a continuous marker to correctly classify a binary outcome; it plots 100-specificity (False positive rate) on the *x*-axis against the sensitivity (True positive rate) on the *y*-axis. The optimum would be 100% specificity (0% false positive rate) and 100% sensitivity (100% true positive rate) which corresponds to the top left corner of the plot. Statistical analyses were conducted using the Stata software (StataCorp LP, Texas, and USA). *p*-values < 0.05 were considered significant.

## Results

### French ***Ae. albopictus*** were less susceptible to Asian than to African ZIKV genotypes

**Figure 1.**
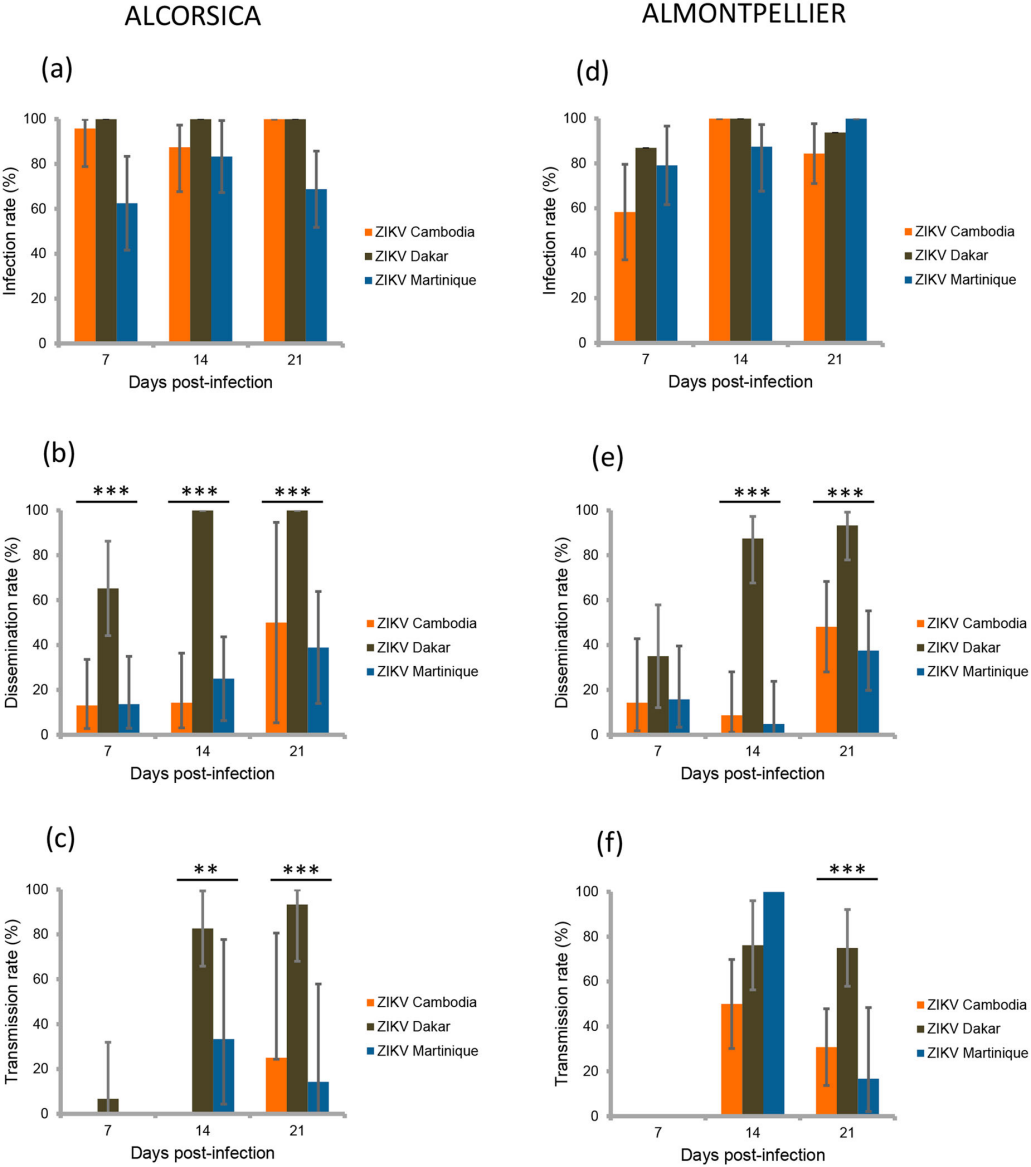
Infection, dissemination and transmission rates of ***Aedes albopictus*** Corsica (a, b, c) and Montpellier (d, e, f), 7, 14, 21 days after exposure to an infectious blood meal containing ZIKV (Cambodia, Dakar and Martinique) provided at a titer of 10^7^ TCID_50_/mL. IR, proportion of mosquitoes with infected body (abdomen plus thorax) among examined mosquitoes; DR, proportion of mosquitoes with virus detected in head among mosquitoes with infected body; TR, proportion of mosquitoes with virus detected in saliva among mosquitoes with infected head. **, *p * 0.01; ***, *p * 0.001.

**Figure 2.**
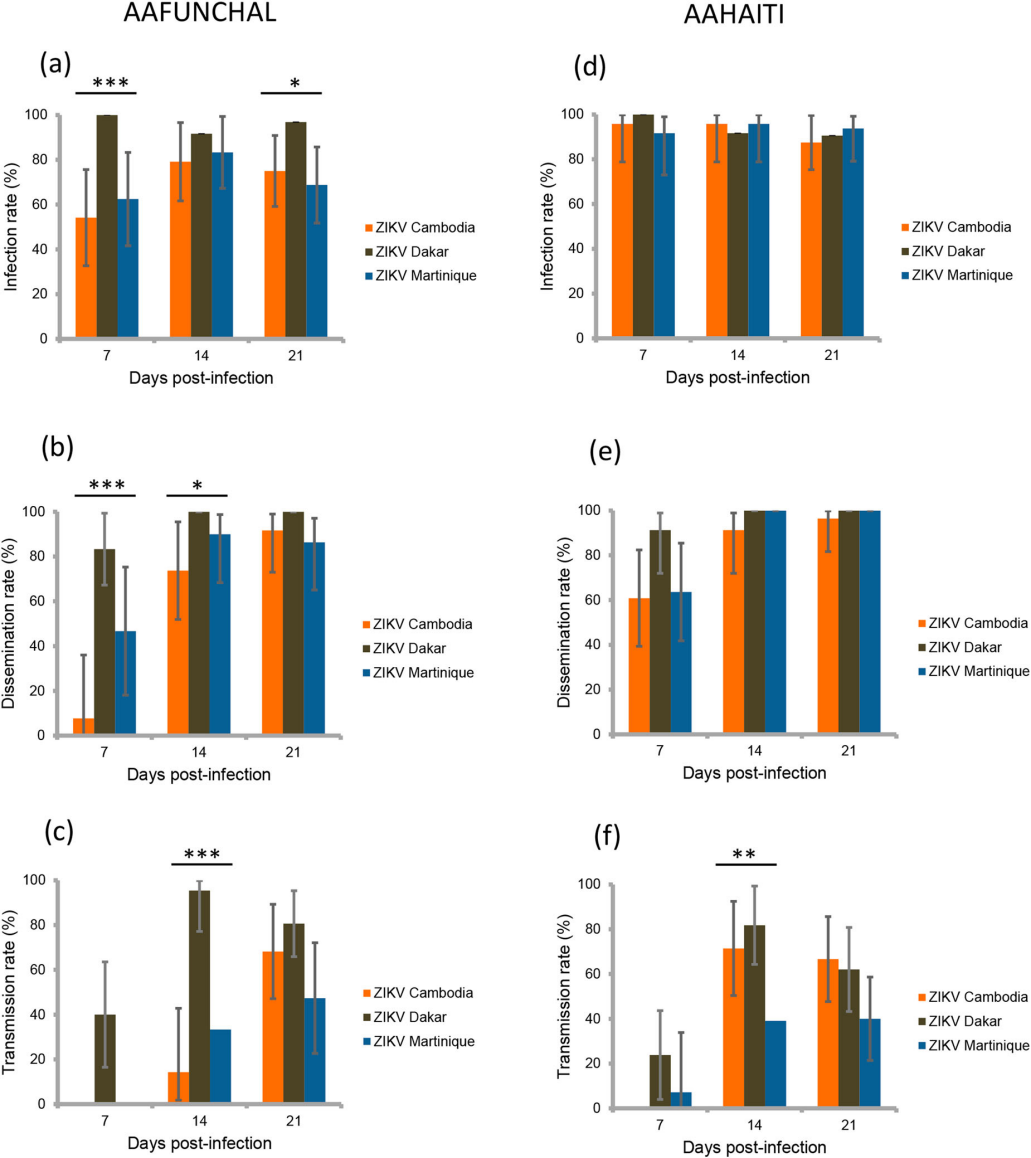
Infection, dissemination and transmission rates of ***Aedes aegypti*** Funchal (a, b, c) and Haiti (d, e, f), 7, 14, 21 days after exposure to an infectious blood meal containing ZIKV (Cambodia, Dakar and Martinique) provided at a titer of 10^7^ TCID_50_/mL. IR, proportion of mosquitoes with infected body (abdomen plus thorax) among examined mosquitoes; DR, proportion of mosquitoes with virus detected in head among mosquitoes with infected body; TR, proportion of mosquitoes with virus detected in saliva among mosquitoes with infected head. *, *p *< 0.05; **, *p * 0.01; ***, *p * 0.001.

Four field-collected populations (two *Ae. albopictus* and two *Ae. aegypti*) were used for experimental infections with ZIKV. Of 903 mosquitoes analysed, 88.6% (800) had infected bodies including the midgut. Whatever the mosquito population and days post-infection (dpi), IR was higher with the ZIKV strain from Dakar than with the two other strains (*p*<0.001) (Table 1 and Figures 1a,d and 2a,d). However, the IR was not similar between mosquito populations; as compared to the *Ae. aegypti* AAFUNCHAL population, the three other populations showed significantly greater IR (*p*<0.001) (Table 1). IR was found to be lower at 7 dpi as compared to later time points (*p*=0.008) (Table 1). No significant interactions between population, dpi and virus, were found.

**Table 1.** Comparison of infection rates between mosquito populations, day post-infection and viral strains (logistic regression model).

		*N*	Infection rate (N)	Adjusted OR (95% CI)	*p*
Population	AAFUNCHAL	240	79.2 (190)	1	< 0.001
	AAHAITI	239	93.6 (223)	3.91 (2.12–7.16)	
	ALCORSICA	184	95.1 (175)	5.46 (2.57–11.63)	
	ALMONTPELLIER	240	88.3 (212)	2.10 (1.25–3.52)	
Day post-infection	7	285	84.6 (241)	0.41 (0.23–0.73)	0.008
	14	286	92.7 (265)	1	
	21	332	88.6 (294)	0.65 (0.37–1.16)	
Virus	Cambodia	295	83.4 (246)	1	< 0.001
	Dakar	299	95.6 (286)	4.59 (2.40–8.77)	
	Martinique	309	86.7 (268)	1.27 (0.80–2.02)	

OR: Odd Ratio, is a measure of association which compares the odds of infection of mosquitoes exposed to the infectious blood meal to the odds of infection of unexposed mosquitoes.

Then, among the 800 infected mosquitoes, 65.4% (523) ensured a successful viral dissemination in mosquito hemocele; dissemination was determined by detecting virus in mosquito head. DR differed by population, *Ae. aegypti* better disseminating ZIKV compared to *Ae. albopictus* (*p*<0.001) (Table 2). An effect of ZIKV strains was evidenced (*p*<0.001), effect that was not the same given the dpi (i.e. interaction between ZIKV strains and dpi) (Table 2). Overall, the ZIKV strain from Dakar caused a significantly higher DR than the two other strains. Moreover, ZIKV Dakar showed an increased DR from 7 dpi which kept increasing with dpi, while the other strains showed low DR at 7 dpi and even 14 and significantly increased at 21 dpi (Table 2 and Figures 1b,e and 2b,e).

**Table 2.** Comparison of dissemination rates between mosquito populations, day post-infection and viral strains (logistic regression model).

		*N*	Dissemination rate (N)	Adjusted OR (95% CI)	*p*
Population	AAFUNCHAL	190	81.0 (154)	1	< 0.001
	AAHAITI	223	90.1 (201)	3.50 (1.80–6.79)	
	ALCORSICA	175	45.1 (79)	0.15 (0.08–0.26)	
	ALMONTPELLIER	212	42.0 (89)	0.07 (0.04–0.13)	
Virus	Cambodia – 7 dpi	73	27.7 (20)	0.23 (0.10–0.55)	< 0.001
	Cambodia – 14 dpi	86	46.5 (40)	1	
	Cambodia – 21 dpi	87	75.9 (66)	5.59 (2.44–12.77)	
			
	Dakar – 7 dpi	90	70.0 (63)	4.06 (1.87–8.80)	
	Dakar – 14 dpi	91	96.7 (88)	83.45 (22.45–310.19)	
	Dakar – 21 dpi	105	98.1 (103)	137.68 (29.45–643.57)	
			
	Martinique – 7 dpi	78	34.6 (27)	0.45 (0.20–1.00)	
	Martinique – 14 dpi	88	54.5 (48)	1.66 (0.78–3.56)	
	Martinique – 21 dpi	102	66.7 (68)	3.63 (1.70–7.76)	

OR: Odd Ratio, is a measure of association which compares the odds of viral dissemination of mosquitoes exposed to the infectious blood meal to the odds of viral dissemination of unexposed mosquitoes.

At the final step, among the 523 mosquitoes with viral dissemination, 50.7% (265) were capable of transmitting the virus. TR was significantly lower at 7 dpi as compared to later time points, even after adjusting for population and ZIKV strains (*p*<0.001; Table 3). Adjusting for dpi, TR differed by viral strains, but the effect of viral strains was not similar in all populations (i.e. interaction between population and viral strain) (Table 3). Overall, the ZIKV strain from Dakar presented the highest TR; TR was not different between AAFUNCHAL and ALCORSICA (*p*=0.16) (Figure 2c,f) but was significantly higher in AAFUNCHAL than in ALMONTPELLIER or AAHAITI (both *p*=0.014) (Figure 1c,f). With the ZIKV strain from Cambodia, as compared to AAHAITI, TR was significantly lower in ALCORSICA and in ALMONTPELLIER (*p*=0.026 and *p*=0.045, respectively). Ultimately, with ZIKV strain from Martinique, we did not evidence TR differences between the four populations.

**Table 3.** Comparison of transmission rates between mosquito populations, day post-infection and viral strains (logistic regression model).

		*N*	Transmission rate (N)	Adjusted OR (95% CI)	*p*
Population – Virus	AAFUNCHAL – Cambodia	37	45.9 (17)	1	< 0.0001
	AAFUNCHAL – Dakar	73	74.0 (54)	10.10 (3.81–26.77)	
	AAFUNCHAL – Martinique	44	34.1 (15)	0.75 (0.30–1.88)	
			
	AAHAITI – Cambodia	62	53.2 (33)	2.19 (0.92–5.22)	
	AAHAITI – Dakar	72	56.9 (41)	3.36 (1.40–8.05)	
	AAHAITI – Martinique	67	32.8 (22)	0.77 (0.33–1.79)	
			
	ALCORSICA – Cambodia	10	10.0 (1)	0.18 (0.02–1.65)	
	ALCORSICA – Dakar	53	64.1 (34)	5.13 (1.93–16.63)	
	ALCORSICA – Martinique	16	18.7 (3)	0.33 (0.08–1.40)	
			
	ALMONTPELLIER – Cambodia	17	29.4 (5)	0.55 (0.16–1.94)	
	ALMONTPELLIER – Dakar	56	66.1 (37)	3.15 (1.29–7.72)	
	ALMONTPELLIER – Martinique	16	18.7 (3)	0.33 (0.80–1.40)	
Dpi	7	110	13.6 (15)	0.05 (0.03–0.11)	< 0.0001
	14	176	62.5 (110)	1	
	21	237	59.1 (140)	1.02 (0.65–1.60)	

OR: Odd Ratio, is a measure of association which compares the odds of viral transmission of mosquitoes exposed to the infectious blood meal to the odds of viral transmission of unexposed mosquitoes.

### Viral dissemination was detected when mosquito bodies (abdomen plus thorax) contain at least 4800 viral copies

**Figure 3.**
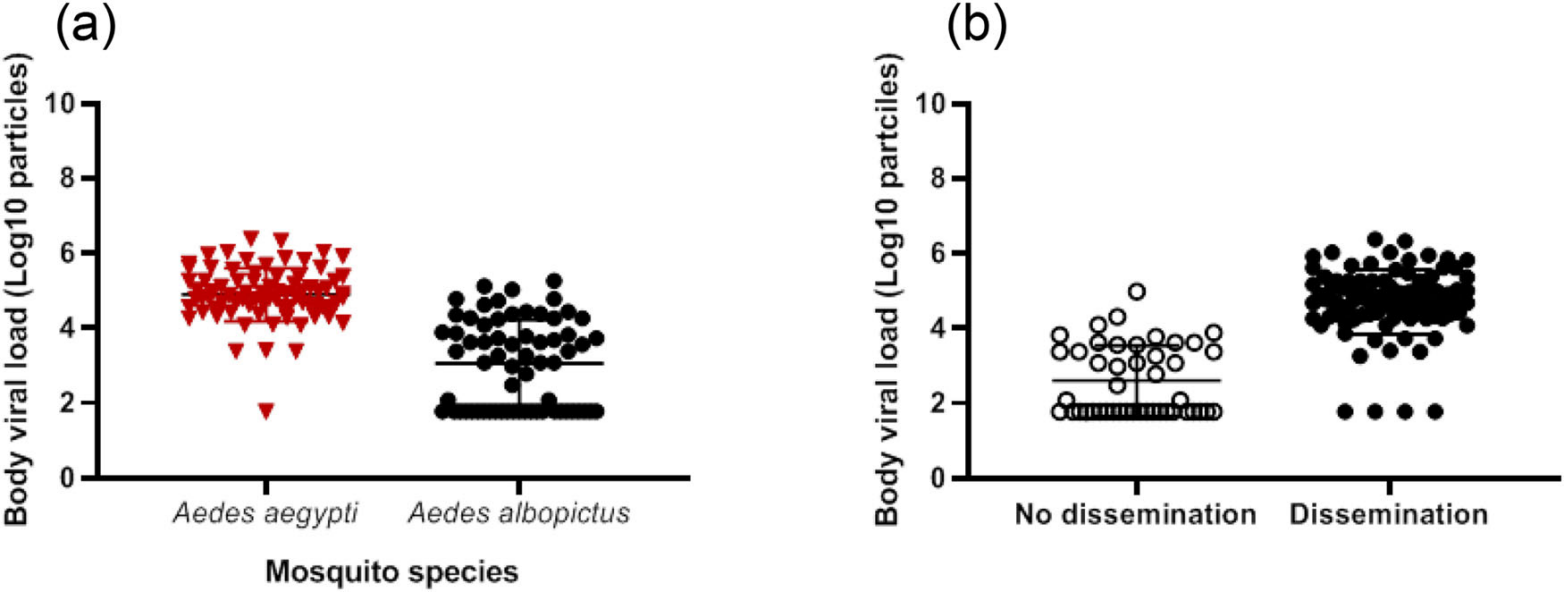
Viral loads in mosquito body according to mosquito species (a) and viral dissemination status (b). Bodies (abdomen plus thorax) were homogenized and supernatants were titrated on Vero cells. Seven days after, viral copies were detected by CPE after staining with crystal violet.

**Figure 4.**
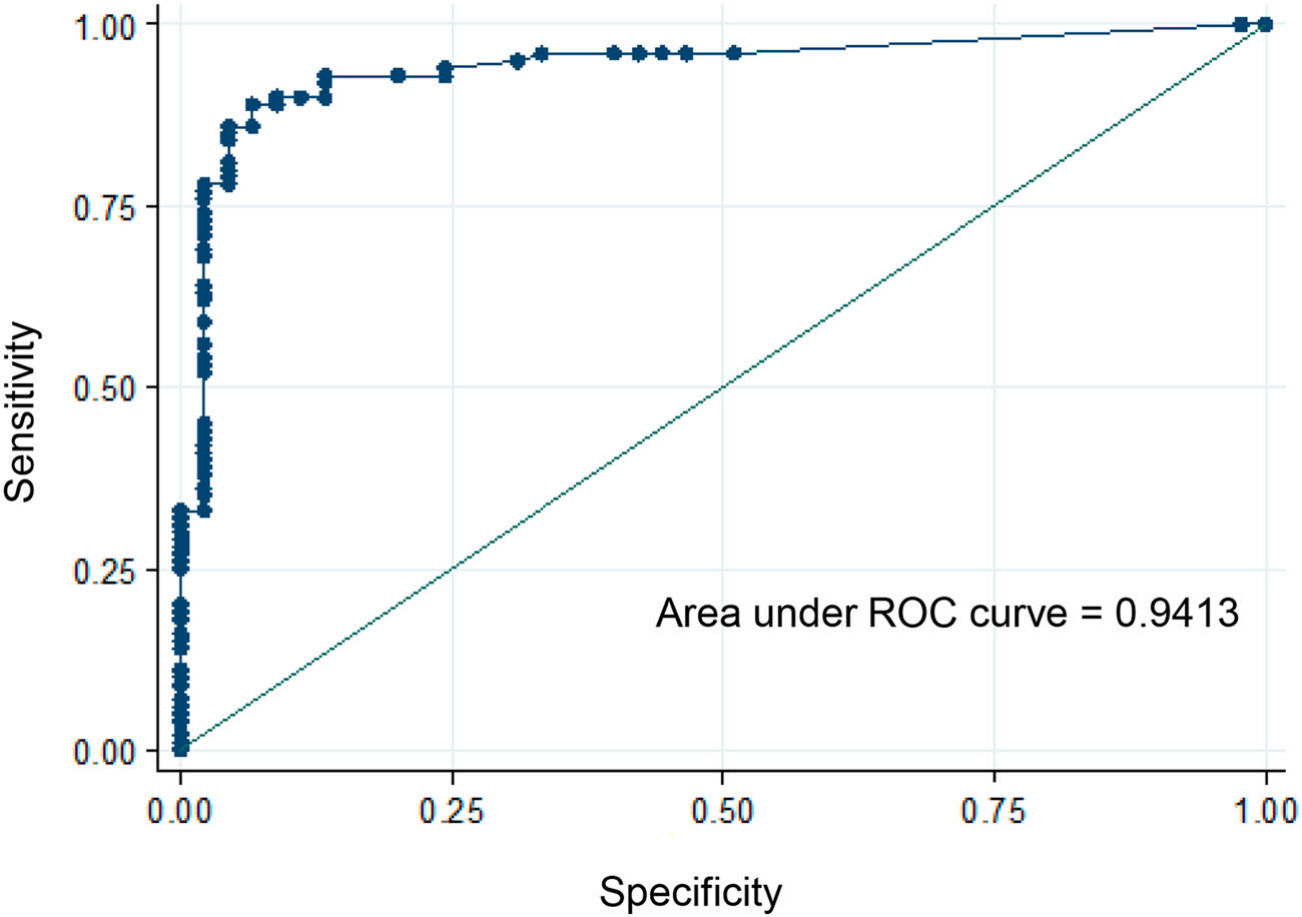
ROC curve to identify mosquitoes capable of disseminating the virus according to the viral load in body.

We selected the 145 mosquitoes that were infected with the ZIKV strain from Martinique which has lastly circulated in the Americas and the Caribbean, and provided estimations of viral loads at each step in mosquitoes: midgut/body infection, dissemination and transmission. The viral load in body was significantly lower in *Ae. albopictus* than in *Ae. aegypti* (mean±SD (N): 3.0 Log_10_±1.2 (66) vs. 4.9 Log_10_±0.7 (79), respectively; *p*<10^−4^) (Figure 3a). The viral load in body was significantly higher in mosquitoes with viral dissemination from the midgut into the hemocele than in those without viral dissemination (4.7 Log_10_±0.9 (100) vs. 2.6 Log_10_±0.9 (45), respectively; *p*<10^−4^) (Figure 3b). To estimate the threshold of viral load in mosquito body to trigger viral dissemination, we pooled all 145 infected mosquitoes and built a ROC curve (Figure 4). The AUC (Area Under the Curve) was calculated; it corresponds to the surface below the ROC curve. The maximum value is 1 and a value over 0.9 is considered very good. We estimated an AUC value of 0.9413 showing a good ability of the viral load level in body to discriminate mosquitoes with and without viral dissemination. We also estimated the threshold which offers the best sensitivity/specificity balance; it maximizes the proportion of “correctly classified individuals”. The number of 4800 viral copies in body was the threshold that correctly categorized the highest proportion of mosquitoes according to their viral dissemination status (Figure 4). Hence, out of 100 mosquitoes with viral dissemination, 93 presented a viral load above this threshold. On the other hand, of 45 mosquitoes without dissemination, 39 had a viral load below the threshold (Supplementary Table 1).

**Figure 5.**
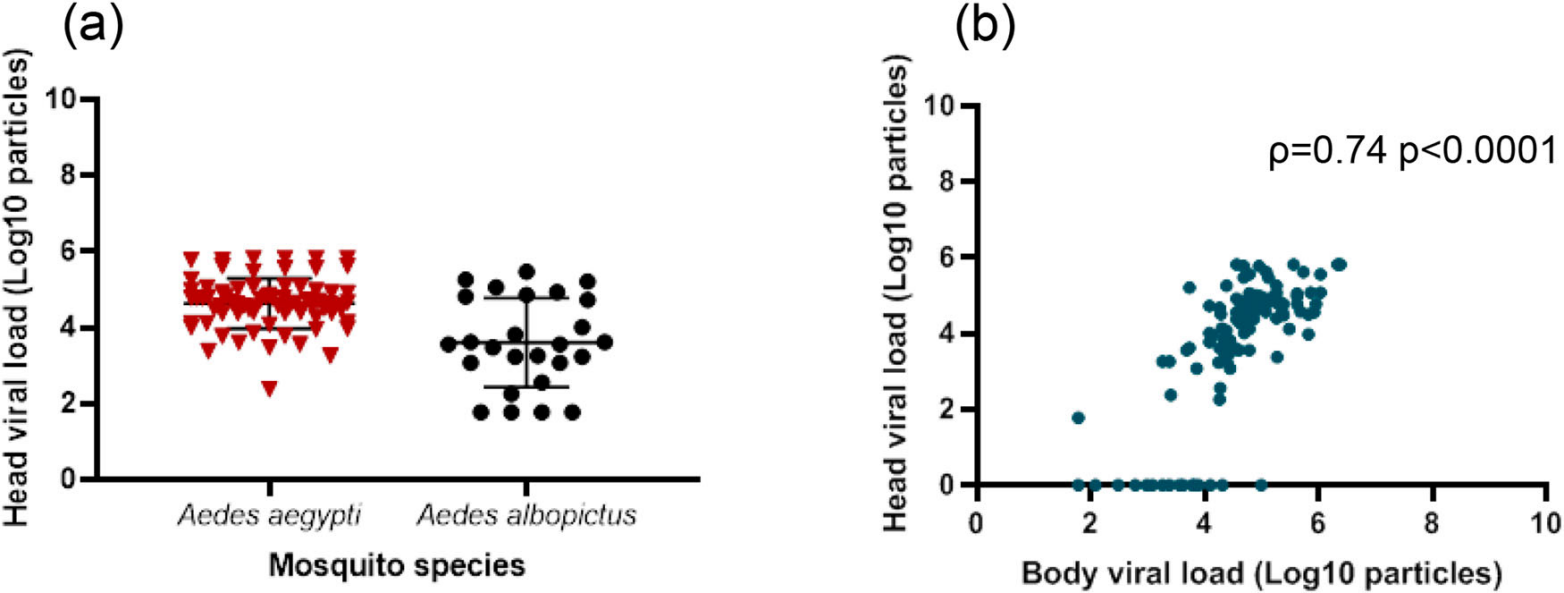
Viral loads in mosquito heads according to mosquito species (a) and correlation between viral loads in bodies and heads (b).

In the 100 mosquitoes with viral dissemination, the viral load in heads was significantly lower in *Ae. albopictus* than in *Ae. aegypti* (3.6 Log_10_±1.2 (26) vs. 4.6 Log_10_±0.7 (74), respectively; *p*<10^−4^) (Figure 5a). A strong correlation was found between the viral loads in bodies and heads (Figure 5b; *ρ*=0.74: *p*<10^−4^).

### Viral transmission was observed when mosquitoes host at least 12,000 viral copies in body

**Figure 6.**
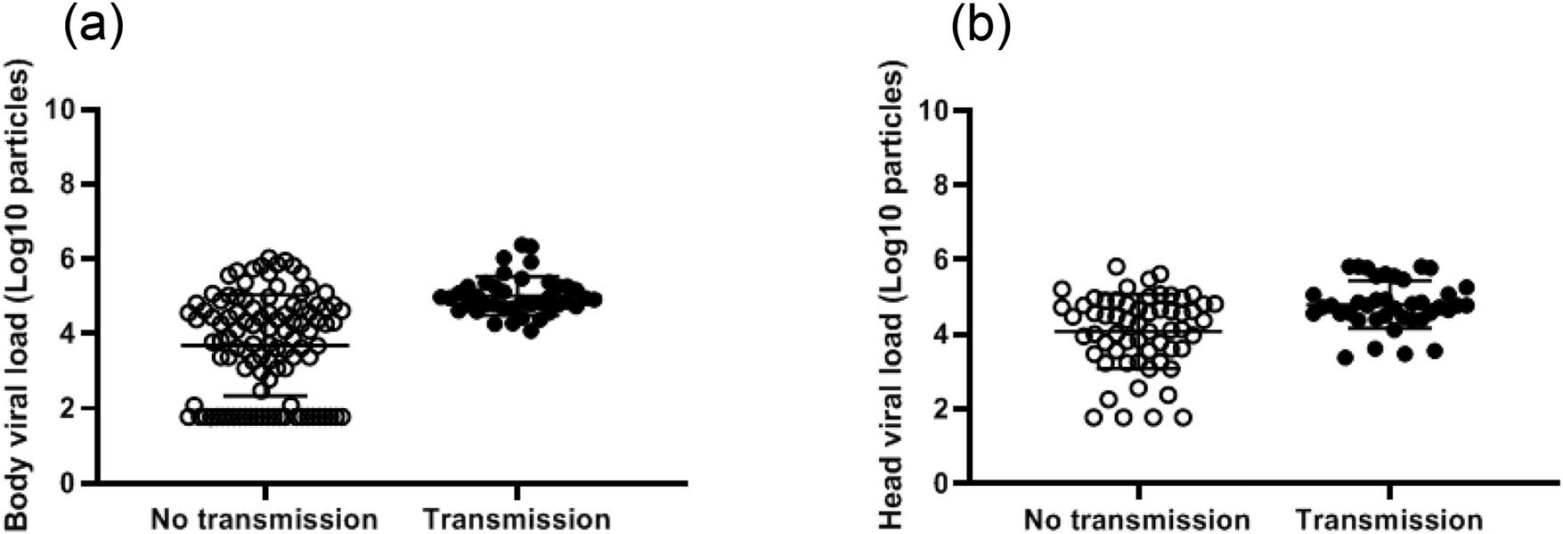
Viral loads in mosquito bodies (a) and heads (b) according to viral transmission status.

**Figure 7.**
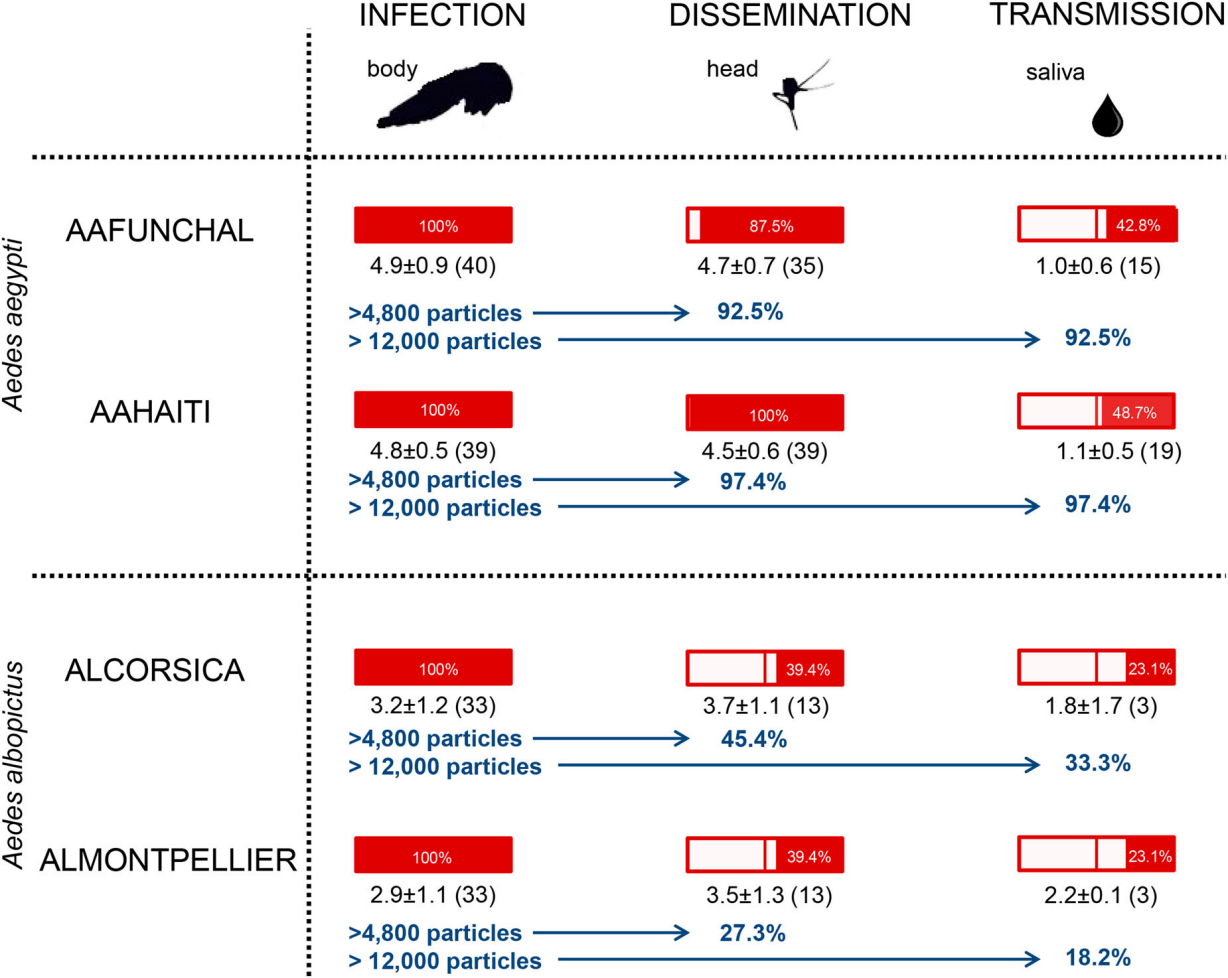
Infection, dissemination and transmission rates of *Aedes aegypti* (AAFUNCHAL and AAHAITI) and *Aedes albopictus* (ALCORSICA and ALMONTPELLIER), 14 days after infection with ZIKV Martinique provided at a titer of 10^7^ TCID_50_/mL. Body **(**Abdomen plus thorax), head and saliva were titrated on Vero cells to evaluate respectively infection, dissemination and transmission rates (rectangle in red). The mean number of viral particles is provided under each rectangle. The proportion of mosquitoes that meet the two criteria: ≥4800 viral copies and ≥12,000 viral copies in body needed to trigger dissemination and transmission respectively, were presented for each mosquito population.

The viral load, measured in body/midguts, was significantly higher in mosquitoes able to transmit than in those not able to transmit (5.0 Log_10_±0.5 (40) vs. 3.7 Log_10_±1.3 (105), respectively; *p*<10^−4^) (Figure 6a). As above, a ROC curve was used to evaluate the capacity of viral load in body to discriminate mosquitoes that could transmit from the others (Supplementary Fig. 1). The area under the curve (AUC: 0.8069) did not reflect a good ability to well discriminate mosquitoes with and without viral transmission based on viral loads in bodies. When using the same threshold as before (i.e. 4800 copies in body), all 40 mosquitoes able to transmit had a viral load above this threshold, but out of 60 mosquitoes unable to transmit, 53 had a viral load above the threshold, meaning that a high midgut infection is a prerequisite for transmission but is not a sufficient criterion alone (Supplementary Table 2). In fact, all mosquitoes able to transmit presented a viral load ≥ 12,000 viral copies in the body. In the 40 mosquitoes with viral transmission, the viral load in saliva was significantly higher in *Ae. albopictus* than in *Ae. aegypti* (respectively 2.0 Log_10_±1.1 (6) vs. 1.1 Log_10_±0.5 (34), when pooling the two *Ae. aegypti* and the two *Ae. albopictus* separately; *p*=0.02) (Figure 7, Supplementary Fig. 2).

### Viral load in heads was not a good indicator of successful transmission

The viral load measured in heads was also found to be significantly higher in the mosquitoes that could transmit compared to those which could not (4.8 Log_10_±0.6 (40) vs. 4.1 Log_10_±1.0 (60), respectively; *p*<10^−3^) (Figure 6b). The area under the ROC curve was used to evaluate the capacity of viral load in head to discriminate mosquitoes that could transmit from the others (AUC: 0.8069; Supplementary Fig. 3); the area under the curve did not reflect a good ability to well discriminate mosquitoes with and without viral transmission based on viral loads in heads. The threshold which correctly classified the largest proportion of mosquitoes was 24,600 viral copies (Supplementary Table 3). When using the same threshold 24,600 copies in mosquito heads, of 40 mosquitoes able to transmit, 34 had a viral load above this threshold and out of 60 mosquitoes unable to transmit, 32 had a viral load below the threshold, meaning that a high viral load in head did not necessarily lead to transmission. When considering another threshold of 2400 viral copies, a viral load ≥ 2400 viral copies in heads was necessary but not sufficient to allow transmission (Supplementary Table 4).

### ***Ae. albopictus*** was less efficient to disseminate and transmit ZIKV

By examining the total of 265 mosquitoes (considering the four populations and three ZIKV strains) able to transmit, viral load in saliva was not correlated with the viral load in body nor in head (*p*>0.05). However, virus was detected in saliva only if viral load was higher than 12,000 viral copies in body (Supplementary Fig. 4a) or higher than 2400 viral copies in head (Supplementary Fig. 4b).

Considering the threshold of 4800 particles in body correlated to an effective dissemination and 12,000 particles in body correlated to an effective transmission, ALMONTPELLIER was the least efficient of the four populations tested; only 27.3% of mosquitoes were able to disseminate and 18.2% to transmit the virus (Figure 7).

## Discussion

Our study indicates that *Ae. albopictus* mosquitoes from France are less able to transmit the Asian genotype of ZIKV which was responsible of the last Zika pandemic, based on two lines of evidence. First, *Ae. albopictus* France (Montpellier and Corsica) were less competent to transmit the two Asian genotypes than the African ZIKV. Second, only a limited percentage of *Ae. albopictus* France met the two main criteria allowing effective viral dissemination and transmission, viral load in body ≥4800 viral copies to allow dissemination and ≥12,000 viral copies to allow transmission.

### Asian ZIKV better transmitted by ***Ae. aegypti*** than ***Ae. albopictus***

First introduced in Funchal [21] in Madeira, an autonomous region of Portugal, *Ae. aegypti* was responsible of the last main outbreak of dengue in the European Union [22]. This mosquito was as efficient as the typical tropical vector *Ae. aegypti* from Haiti to transmit all three ZIKV (Cambodia, Dakar and Martinique) in addition to be experimentally able to transmit dengue virus (DENV) and chikungunya virus (CHIKV) [23]. On the other hand, *Ae. albopictus* is well established in Europe since 1990 [14] and in France since 2004 [24]. *Ae. albopictus* from Corsica and Montpellier were less efficient to disseminate the 3 ZIKV strains than *Ae. aegypti* underlining a strong barrier limiting viral dissemination from the midgut and accordingly, a low viral transmission [17].

In a more global way, ZIKV Dakar was better disseminated and transmitted by both species. The first evidence of ZIKV circulation was reported in Africa [3] and in Senegal in 1962 [25]. Since then, ZIKV was periodically notified in West Africa which experienced several independent introductions of ZIKV strains during the last century [26]. Surprisingly, up to date, no reports on ZIKV strains exported from West Africa were mentioned. In Europe, viremic travellers returning from regions endemic for ZIKV were frequently reported, becoming then a potential source of local transmission by *Ae. albopictus*. It is tempting to speculate that *Ae. albopictus* France will be able to sustain a local transmission of ZIKV if the viral strain comes from West Africa; a same scenario was lastly observed for CHIKV [27].

### A threshold is needed to initiate dissemination and transmission of ZIKV in ***Aedes*** mosquitoes

While it is admitted that to infect vectors, a minimum level of host viremia is necessary [28], only few studies are available on thresholds in vectors needed to trigger the successive steps leading to transmission with virus excreted from mosquito saliva [16,29–31]. Therefore, we measured thresholds of viral loads in the mosquito body to trigger dissemination and transmission of ZIKV Martinique, which circulated in the Caribbean in 2015 [32]. *Ae. aegypti* presented a higher viral load in body compared to *Ae. albopictus* and mosquitoes able to disseminate the virus harbored a higher viral load in body suggesting a threshold to initiate viral dissemination. A threshold of 4800 viral copies in the body permits viral dissemination to 92.5–97.4% of *Ae. aegypti* mosquitoes and to only 27.3–45.4% of *Ae. albopictus* (see Figure 7). Considering the viral loads measured in humans (i.e. 5.36 Log10 RNA copies [33]), mosquitoes by ingesting 2–3 µL of blood [34] absorb 400–700 RNA copies. Since, on the one hand, mosquitoes were allowed to ingest around 30,000 viral copies (3 µL of infectious blood provided at a titer of 10^7^ TCID_50_/mL), and on the other hand, only 18.2% of *Ae. albopictus* mosquitoes that received this inoculum were able to transmit ZIKV, it is obvious that the actual capability of European *Ae. albopictus* to become infected and initiate transmission of ZIKV after the bite on a viremic patient is extremely low. The midgut of *Ae. albopictus* is clearly very efficient to limit ZIKV dissemination and transmission [17]. In addition to the viral load, the viral genotype would also play a role as demonstrated for CHIKV [35]; anatomical barriers such as the midgut and salivary glands may contribute to select variants with a higher potential to cause outbreaks.

In conclusion, our study highlights that the different ZIKV genotypes were not equally transmitted by *Ae. aegypti* and *Ae. albopictus*. ZIKV from Dakar of the West African genotype was better transmitted by both species with, however, a higher transmission potential for *Ae. aegypti* compared to *Ae. albopictus*. At a threshold of 12,000 viral copies in mosquito body, less than 34% of *Ae. albopictus* were able to transmit, stressing out the low vector competence of French *Ae. albopictus* for the ZIKV responsible for the last Zika pandemic. We have proposed a valuable tool to predict viral dissemination and transmission in *Aedes* mosquitoes based on the threshold response where all mosquitoes are able to transmit above a threshold of viremia in human cases. This model can be used to infer the proportion of mosquitoes contributing to the transmission of an arbovirus, and to build maps to detail global ZIKV transmission risk [36]. This assessment should be extended to other mosquito populations as the outcome of infection depends on three-way combination of mosquito population, virus genotype and environmental factors [37].

## Supplementary Material

Supplemental Material
