# Supplementary material for: Zika virus threshold determines transmission by European Aedes albopictus mosquitoes
Source: Emerg Microbes Infect. 2019 Nov 18;8(1):1668–78. doi: 10.1080/22221751.2019.1689797 (PMC6882490; doi:10.1080/22221751.2019.1689797)

**Supplementary Figure 1.** ROC curve to identify mosquitoes capable of transmitting the virus according to viral loads in bodies.

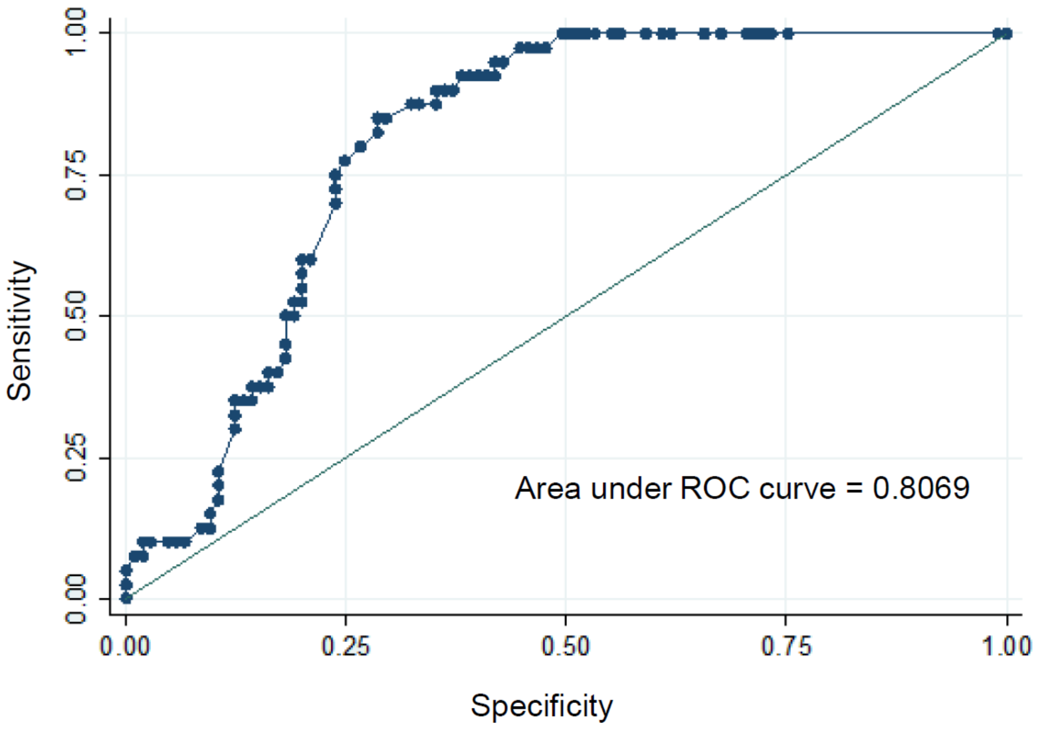

Supplement: Supplemental Material [file TEMI_A_1689797_SM7565.zip › Figure_S1.pdf]
