# Supplementary material for: Zika virus threshold determines transmission by European Aedes albopictus mosquitoes
Source: Emerg Microbes Infect. 2019 Nov 18;8(1):1668–78. doi: 10.1080/22221751.2019.1689797 (PMC6882490; doi:10.1080/22221751.2019.1689797)

Supplementary Figure 3. ROC curve to identify mosquitoes capable of transmitting the virus according to viral loads in heads.

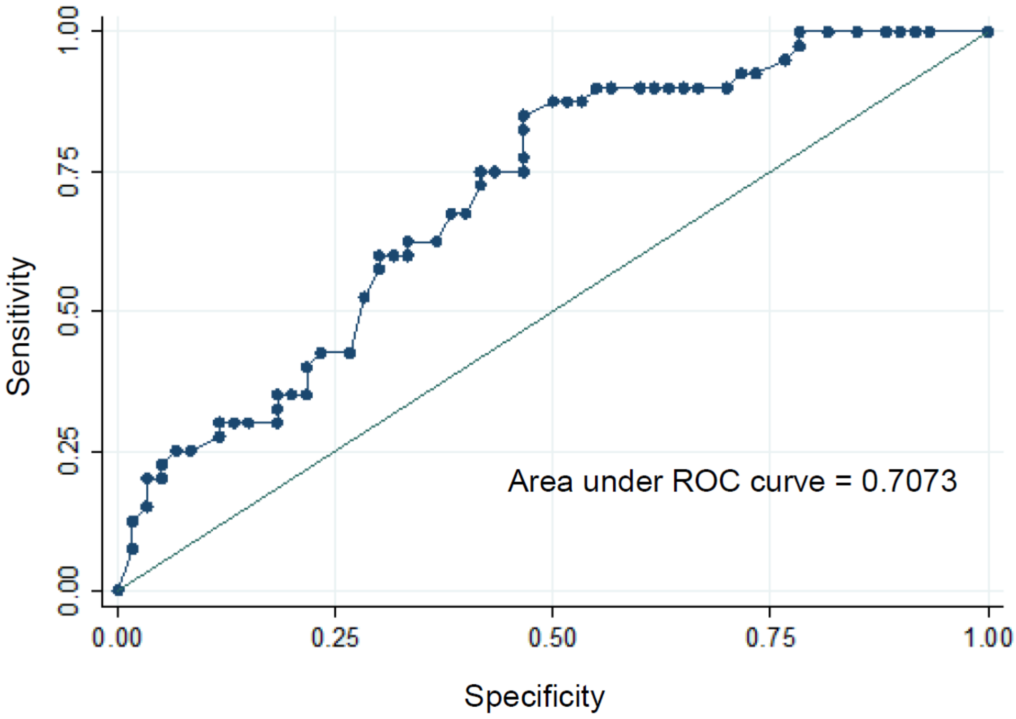

Supplement: Supplemental Material [file TEMI_A_1689797_SM7565.zip › Figure_S3.pdf]
