# Supplementary material for: Zika virus threshold determines transmission by European Aedes albopictus mosquitoes
Source: Emerg Microbes Infect. 2019 Nov 18;8(1):1668–78. doi: 10.1080/22221751.2019.1689797 (PMC6882490; doi:10.1080/22221751.2019.1689797)

Supplementary Figure 4. Correlation between viral loads in bodies and saliva (a), and between viral loads in heads and saliva (b). Virus in saliva was detected only if viral loads were higher than 4 Log in bodies and 3.4 Log in heads.

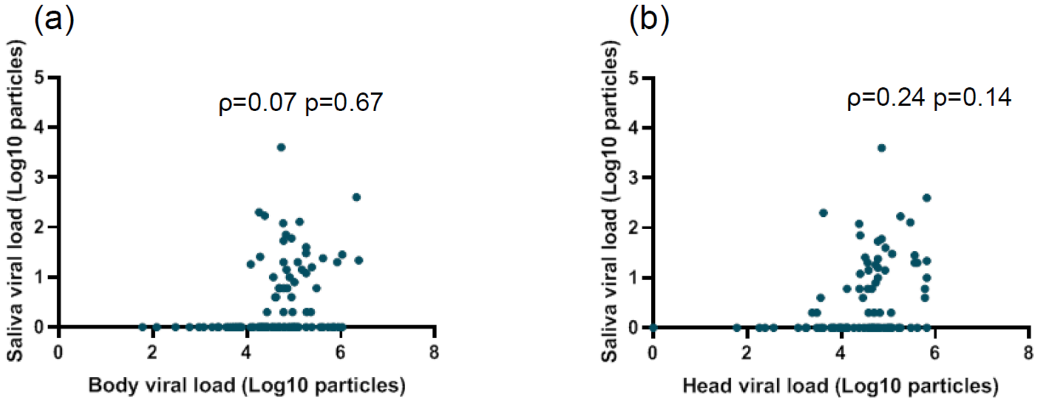

Supplement: Supplemental Material [file TEMI_A_1689797_SM7565.zip › Figure_S4.pdf]
