# Supplementary figures and images for: Zika virus threshold determines transmission by European Aedes albopictus mosquitoes
Source: Emerg Microbes Infect. 2019 Nov 18;8(1):1668–78. doi: 10.1080/22221751.2019.1689797 (PMC6882490; doi:10.1080/22221751.2019.1689797)

1 **Supplementary Figure 2.** Viral loads in mosquito saliva according to mosquito  
2 species.

3

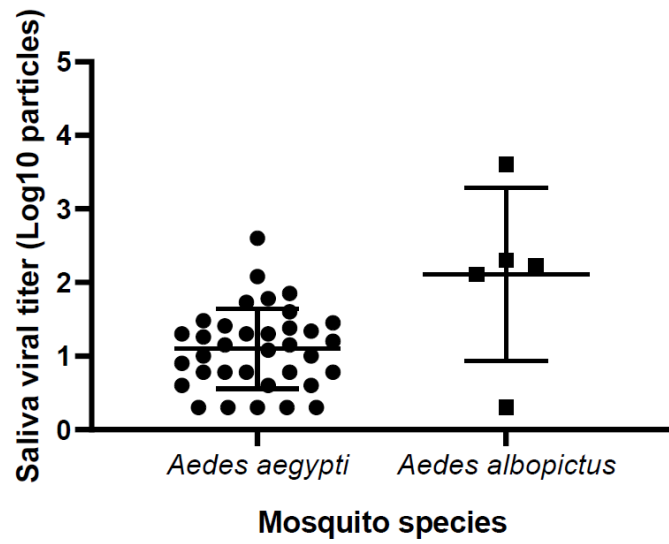

4

Supplement: Supplemental Material [file TEMI_A_1689797_SM7565.zip › Figure_S2.pdf]
