# Supplementary material for: Zika virus threshold determines transmission by European Aedes albopictus mosquitoes
Source: Emerg Microbes Infect. 2019 Nov 18;8(1):1668–78. doi: 10.1080/22221751.2019.1689797 (PMC6882490; doi:10.1080/22221751.2019.1689797)
Supplement: Supplemental Material [file TEMI_A_1689797_SM7565.zip › Table_S1_final.doc]

**Supplementary Table 1.** Viral loads (VL) measured in midguts to predict dissemination.

|  | Dissemination | No dissemination | Total |
| --- | --- | --- | --- |
| VL ≥ 4,800 copies | 93 | 6 | 99 |
| VL< 4,800 copies | 7 | 39 | 46 |
| Total | 100 | 45 | 145 |
