# Supplementary material for: Zika virus threshold determines transmission by European Aedes albopictus mosquitoes
Source: Emerg Microbes Infect. 2019 Nov 18;8(1):1668–78. doi: 10.1080/22221751.2019.1689797 (PMC6882490; doi:10.1080/22221751.2019.1689797)
Supplement: Supplemental Material [file TEMI_A_1689797_SM7565.zip › Table_S3_final.doc]

**Supplementary Table 3.** Viral loads (VL) measured in heads to predict transmission at a threshold of 24,600 viral copies.

|  | Transmission | No transmission | Total |
| --- | --- | --- | --- |
| VL ≥ 24,600 copies | 34 | 28 | 72 |
| VL< 24,600 copies | 6 | 32 | 38 |
| Total | 40 | 60 | 100 |
